# Proficiency testing in immunohistochemistry—experiences from Nordic Immunohistochemical Quality Control (NordiQC)

**DOI:** 10.1007/s00428-015-1829-1

**Published:** 2015-08-26

**Authors:** Mogens Vyberg, Søren Nielsen

**Affiliations:** NordiQC, Institute of Pathology, Aalborg University Hospital, Aalborg, Denmark; Department of Clinical Medicine, Aalborg University, Aalborg, Denmark

**Keywords:** Immunohistochemistry, NordiQC, External quality assurance

## Abstract

Despite extensive use of immunohistochemistry (IHC) for decades, lack of standardization remains a major problem, even aggravated in the era of targeted therapy. Nordic Immunohistochemical Quality Control (NordiQC) is an international academic proficiency testing (PT) program established in 2003 primarily aimed at assessing the analytical phases of the laboratory IHC quality. About 700 laboratories from 80 countries are currently participating. More than 30,000 IHC slides have been evaluated during 2003–2015. Overall, about 20 % of the staining results in the breast cancer IHC module and about 30 % in the general module have been assessed as insufficient for diagnostic use. The most common causes for insufficient results are less successful antibodies (poor and less robust antibodies, poorly calibrated ready-to-use (RTU) products, and stainer platform-dependent antibodies; 17 %), insufficiently calibrated antibody dilutions (20 %), insufficient or erroneous epitope retrieval (27 %), less sensitive visualization systems (19 %), and other (heat- and proteolysis-induced impaired morphology, endogenous biotin reaction, drying out phenomena, stainer platform-dependant protocol issues; 17 %). Approximately, 90 % of the insufficient results are characterized by either a too weak or false negative staining, whereas in the remaining 10 %, a poor signal-to-noise ratio or false positive staining is seen. Individually tailored recommendations for protocol optimization and identification of best tissue controls to ensure appropriate calibration of the IHC assay have for many markers improved IHC staining as well as inter-laboratory consistency of the IHC results. RTUs will not always provide an optimal result and data sheets frequently misguide the laboratories hampering the improvement in IHC quality. The overall data generated by NordiQC during 12 years indicates that continuous PT is valuable and necessary. Detailed description of the results of the NordiQC programme is available on www.nordiqc.org and summarized in this paper.

## Introduction

Immunohistochemistry is technically complex, and no aspect of this complexity can be ignored, from the moment of collecting the specimen to issuance of the final report [1].

During the last four decades in pathology, immunohistochemistry (IHC) has developed into an indispensable ancillary diagnostic tool (class I assay), particularly in the classification of neoplastic lesions. In the era of targeted cancer therapy, IHC has also become a companion diagnostic (class II assay). However, while the potential of IHC in pathology is universally accepted, it is still considered a “special stain” developed in the individual laboratory rather than a tissue-based qualitative or quantitative immunoassay, and its reliability is compromised by lack of standardization, causing a high risk of suboptimal laboratory performance which leads to inferior pathology diagnostics.

The “total test approach,” including standardization of the preanalytical, analytical, and postanalytical processes, is of outmost importance to ensure the technical, diagnostic, and clinical quality of IHC. Nevertheless, numerous steps in the tissue processing and staining protocol are still defined by the individual laboratory, the selection of tissue controls is largely unregulated, and the interpretation of the staining results partly subjective. Internal quality control is focused on the consistency of the IHC assays, but does generally not give information about the technical or diagnostic quality, and insufficient assays often pass unnoticed through the laboratory validation because of improper control tissues or lack of knowledge about reaction patterns.

In selected areas, recommendations for standardization of class II assays have been published by working groups and ad hoc committees [2–5]. While these are helpful, many issues remain. In particular, the identification of the best antibodies and protocols for the IHC assays is still a challenge for the individual laboratory. For each epitope to be demonstrated, numerous parameters influence the sensitivity and specificity of the assay. Even though meticulous and methodological technical calibration of the IHC assays might be performed in the laboratory, and a comprehensive quality control system might exist to monitor the consistency of the assay, it is still difficult to evaluate whether the IHC results are at the level expected and comparable to that obtained by other laboratories.

In IHC, proficiency testing (PT) or external quality assurance (EQA) is a method primarily aimed at the analytical outcome, i.e., the staining results, based on circulation of serial sections of multi tissue arrays (TMAs) of “standard processed” tissues to be stained for defined proteins in a large number of laboratories and assessed by a group of experienced pathologists and biomedical scientists. The principal advantage of EQA is the ability to detect differences of staining quality and relate these to the antibodies, protocol parameters, and stainer platforms in order to identify which elements may give sufficient or insufficient staining results. Thus, EQA can provide guidance on how to achieve the best IHC standards. Guidance may be given directly to the participants and used in publications on websites and in scientific journals.

The aim of the present paper is to give a short description of the Nordic immunohistochemical Quality Control (NordiQC) PT scheme.

## Material and methods

In 1999, pathologists from Denmark, Finland, Norway, and Sweden constructed a Nordic pilot scheme for EQA, and by 1st of January 2003, NordiQC was established as an academic PT programme at the Institute of Pathology, Aalborg University Hospital, Aalborg, Denmark. In the first annual scheme, about 70 Nordic laboratories participated. Hereafter, the programme was opened for other countries, and by 2015, more than 700 laboratories from about 80 countries are enrolled. Detailed description of the organization is available on www.nordiqc.org and summarized below.

The NordiQC EQA scheme consists of three modules: (1) general module that includes tests for the most common epitopes demonstrated in surgical and clinical pathology to identify and subclassify neoplasms being performed in three runs per year, each comprising 5–6 tests; (2) breast cancer IHC module that includes tests for HER2, hormone receptors, and other markers relevant in breast cancer pathology being performed in two runs per year; and (3) breast cancer HER2 in situ hybridization (ISH) module, also in two runs per year. Slides of TMAs from standard processed formalin-fixed, paraffin-embedded (FFPE) material are used for all tests. For the breast cancer module and HER2 ISH module, the tissues have been fixed and processed according to the recommendations of the American Society of Clinical Oncology/College of American Pathologists (ASCP/CAP) and ad hoc committees for IHC standardization [2–5]. The TMAs for each epitope typically include 5–6 tissue cores of 4 mm from relevant normal and neoplastic tissues expressing (whenever possible) high, intermediate, and low levels of the epitope to be tested, as well as tissue without epitope expression, in order to evaluate both the level of sensitivity and specificity of the assays. Normal tissues are included to provide essential information about the optimal staining patterns that may allow them to be used by the laboratories as reliable tissue controls. All tissues are evaluated for the target epitope both before and after the TMA construction to ensure that the levels of expression are as expected. Serial 3–4 μm sections are cut, and for each TMA, the target epitope is evaluated in the first, middle, and last section to monitor its expression throughout the tissue. Participants enroll by completing a web-based questionnaire that details about 50 analytical variables regarding antibody, protocol, and platform used for each of the epitopes to be demonstrated. For all tests, two unstained slides are circulated to each of the attending laboratories. The returned stained slides are assessed using a traditional expert panel-based qualitative assessment system. Each staining is evaluated by consensus as optimal, good, borderline, or poor (Table 1). The staining results are correlated to the data submitted by the participants in order to identify variables important to the staining reactions. The general staining results and results of the data analysis are posted on www.nordiqc.org together with information about the required staining patterns of tissues included in the TMAs. Examples of antibodies, protocols, and platforms giving optimal results are also posted. Individual assessment scores are communicated directly by e-mail to the participating laboratories. In case of an insufficient result, tailored recommendations for improvement are given. Reassessments may be requested based on staining of new slides. The impact of recommendations in case of insufficient stains is evaluated based on individual reassessments and repeated challenges for the same epitope.

**Table 1:** NordiQC scoring criteria. Consensus scoring of circulated TMA slides in the assessor board based on the staining quality, i.e., staining intensity in cells expected to be demonstrated, signal-to-noise ratio, background staining, aberrant staining pattern, counterstaining, and preservation of morphology

Score	Criteria
Optimal	Staining reaction considered perfect or close to perfect in all of the included tissue cores.
Good	Staining reaction considered fully acceptable in all of the included tissue cores. However, the protocol may be optimized to ensure the best staining intensity and signal-to-noise ratio.
Borderline	Staining considered insufficient because of, e.g., a generally too weak staining or a false negative staining of one of the included tissues, or a minor false positive staining reaction.
Poor	Staining considered very insufficient because of, e.g., false negative staining of several of the included tissues, or a major false positive staining reaction.

## Results

Detailed description of the NordiQC results is available on www.nordiqc.org and summarized with examples below.

During January 2003–July 2015, 37 assessment runs have been performed in the general IHC module, and 18 in the breast cancer IHC module. Overall, challenges for 89 IHC epitopes have been performed up to 16 times (Table 2). Furthermore, seven runs have been performed in the HER2 ISH module (not included in this paper). The total number of IHC slides assessed exceeds 30,000.

**Table 2:** IHC markers included in NordiQC runs 2003–2015

Alpha-methylacyl-CoA racemase (AMACR)	CyclinD1 (CyD1)	MLH1
Alpha-smooth muscle actin (ASMA)	Cytokeratin (CK) 5	MSH2
Anaplastic lymphoma kinase (ALK) Lymphoma Lung adenocarcinoma	CK 7 CK 19 CK 20	MSH6 Multiple myeloma oncogene 1 (MUM1) Myosin, smooth muscle heavy chain (SMHCM)
B cell specific activator protein (BSAP, Pax5)	CK, high molecular weight	Napsin A
bcl-2protein	CK, low molecular weight	Neurofilament protein (NFP)
bcl-6protein	CK, pan-	Octamer transcription factor-3/4 (OCT3/4)
Calretinin	Desmin	p16^ink4a^
Cancer antigen 125 (CA125)	Detected on GIST-1 (DOG1, anoctamin-1)	p40
Carcinoembryonic antigen (CEA)	E-cadherin	p53
CD3	Epithelial cell adhesion molecule (EpCAM)	p57
CD4	Epithelial membrane antigen (EMA)	p63
CD5	Estrogen receptor alpha (ER)	Paired box gene-2 protein (PAX2)
CD8	Factor VIII related antigen	Paired box gene-8 protein (PAX8)
CD10	GATA3	Placental alkaline phosphatase (PLAP)
CD14	Glial fibrillary acidic protein (GFAP)	PMS2
CD15	Glypican 3	Podoplanin
CD19	Gross cystic disease fluid protein-15 (GCDFP15)	Prostate specific acid phosphatase (PSAP)
CD20 CD23 CD30	HER-2 Breast carcinoma Gastric carcinoma	Prostate-specific antigen (PSA) Prostein Progesterone receptor (PR)
CD31	Hepatocyte antigen (HEPPAR1)	S-100 protein beta
CD34	Human chorionic gonadotropin (HCG)	Sal-like protein 4 (SALL4)
CD45	Immunoglobulin kappa (IgK)	SOX10
CD56	Immunoglobulin lambda (IgL)	Synaptophysin
CD68	Immunoglobulin M (IgM)	Terminal deoxynucleotidyl ransferase (TdT)
CD79a	Ki-67	Vimentin
CD99 CD117 Chromogranin (CGA)	Mammaglobin Melan-A Melanosoma specific antigen (MSA, HMB45)	Wilm’s tumor-1 protein (WT1)

**Fig. 1:**
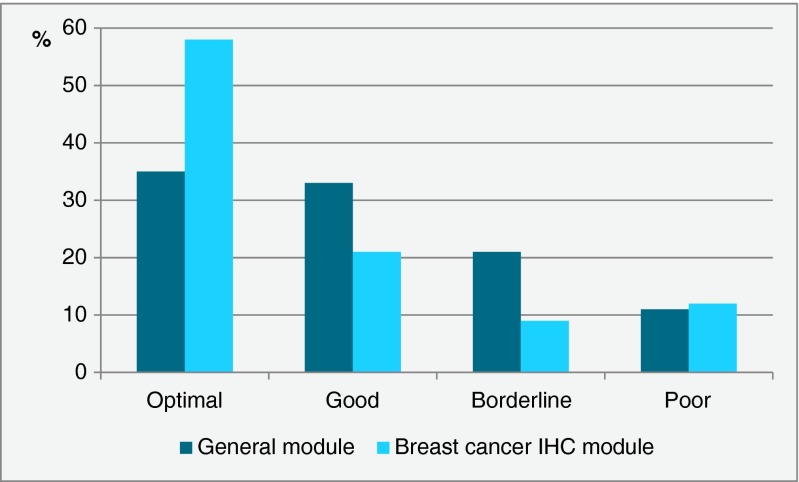
Proportion of assessment scores applied to more than 20,000 assays in the general module and more than 9000 assays in the breast cancer IHC module

In the breast cancer IHC module, around 20 % of all stains assessed by NordiQC have been marked insufficient, i.e., borderline or poor, while in the general module, the proportion of insufficient stains is around 30 % (Fig. 1). In the large majority of about 9000 insufficient assays, the major cause has been identified (Table 3). About 90 % of the insufficient stains are characterized by a too weak or even false negative staining reaction in one or more cores, while the remaining are insufficient due to poor signal-to-noise ratio, false positive, or combined false negative and false positive.

**Table 3:** Major causes of insufficient staining reactions

1. Less successful antibodies (17 %)
a. Poor antibodies^a^
b. Less robust antibodies^b^
c. Poorly calibrated RTUs
d. Stainer platform dependent antibodies
2. Insufficiently calibrated antibody dilutions (20 %)
3. Insufficient or erroneous epitope retrieval (27 %)
4. Error-prone or less sensitive visualization systems^c^ (19 %)
5 Other (17 %)
a. Heat-induced impaired morphology
b. Proteolysis induced impaired morphology
c. Drying out phenomena
d. Stainer platform-dependant protocol issues
e. Excessive counterstaining impairing interpretation

^a^Consistently gives false negative or false positive staining or a poor signal-to-noise ratio in one or more assessment runs

^b^Frequently giving inferior staining results, e.g., due to mouse-anti-Golgi reactions or sensitive to standard operations as blocking of endogenous peroxidase

^c^Biotin-based detection kit for cytoplasmic epitopes, use of detection kits providing a too low sensitivity, or use of detection kits and chromogens giving imprecise localization of the staining signals complicating the interpretation

**Fig. 2:**
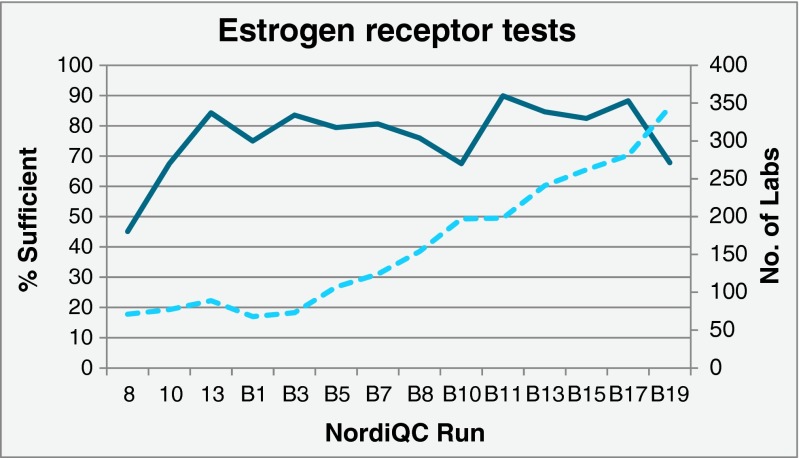
Proportion of sufficient estrogen receptor test results in 14 runs (*full line*) and number of laboratories participating in the challenges (*dotted line*)

**Fig. 3:**
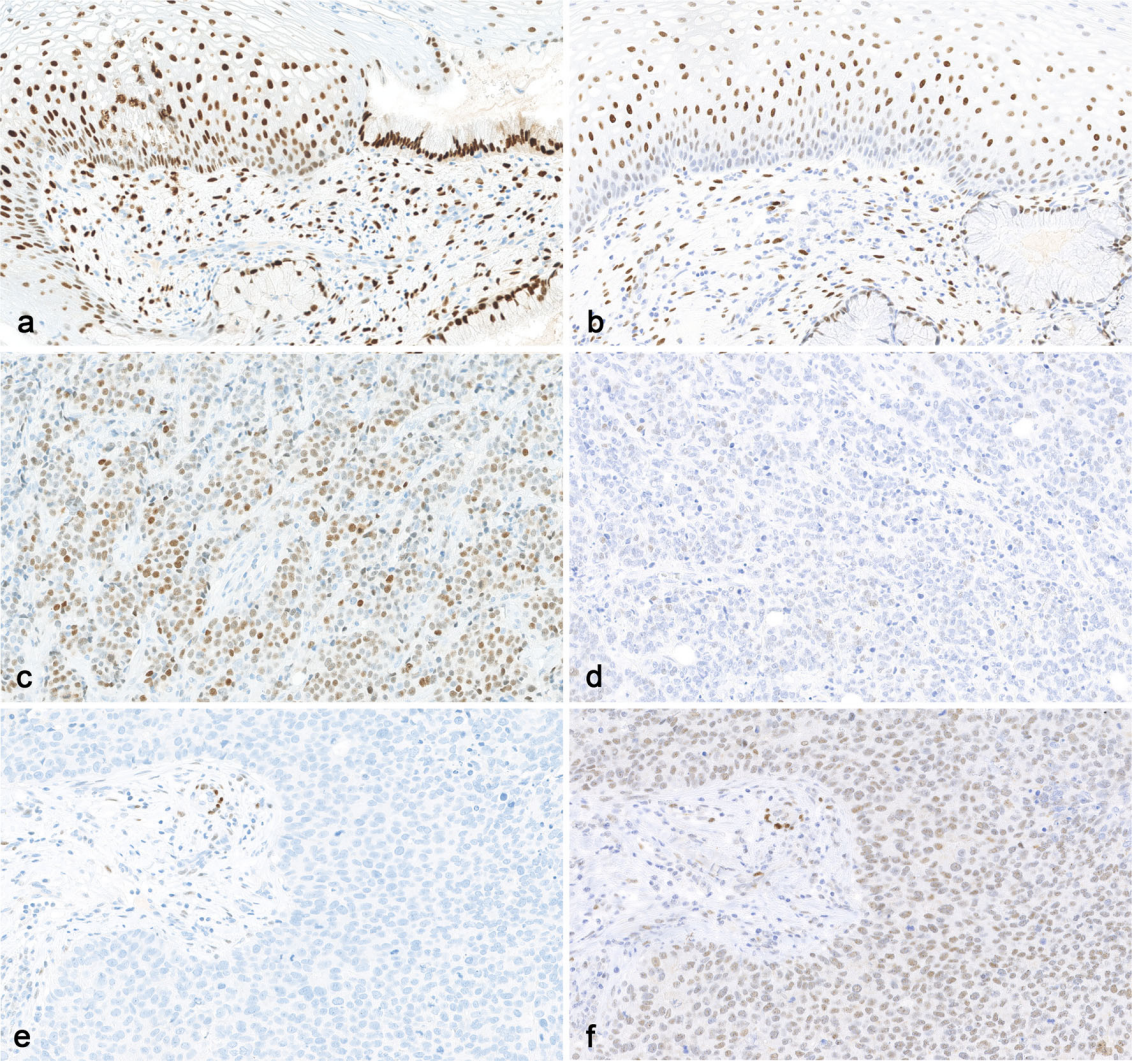
Staining for estrogen receptor. **a** Optimal staining of uterine cervix, which is recommended as positive control tissue. Note moderate staining of the basal squamous epithelial cells, which are low expressors. **b** Insufficient staining of the uterine cervix, the basal cells are negative. This is typically caused by too low antibody titre antibody and/or insufficient HIER. **c** Optimal staining of ductal breast carcinoma; most nuclei are moderately positive. **d** Insufficient staining of same ductal breast carcinoma as in (**c**), based on the same protocol as in (**b**), the tumor is false negative. **e** Optimal staining of an estrogen receptor negative ductal breast carcinoma obtained in all of 225 laboratories using clone SP1, EP1, or 1D5, and 18 out of 37 laboratories using clone 6F11. All neoplastic cells are negative while stromal cells are positive, serving as internal control. **f** False positive staining reaction of the same tumor as in (**e**) obtained in 15 out of 37 laboratories using clone 6F11. This (rare) staining reaction is possibly due to inadequate buffer wash in combination with use of very sensitive protocol (×200)

Tests for estrogen receptor (ER) have been included in 14 runs during 2003–2015. During this period, the proportion of sufficient stains has increased from 45 % in the first run to about 70–90 % in the later runs (Fig. 2), tending to decrease each time a larger group of laboratories participate for the first time. In line with this observation, the pass rate has for “old” participants consistently been higher than for the new ones, in the latest run 73 vs. 51 %. During the same period, marked improvements in the standardization and sensitivity of the protocols for ER have been recorded (see Table 4). Examples of optimal and insufficient ER stains are illustrated in Fig. 3.

**Table 4:** Increasing standardization in staining for estrogen receptor among NordiQC participants

	2003/2008	2015
Ready-to-use antibody/system	17 %^a^	66 %
Commercially available HIER buffer	12 %^b^	94 %
Alkaline HIER buffer	70 %^b^	94 %
Polymer/multimer-based detection kit	56 %^b^	93 %
Fully automated stainer platform	6 %^b^	59 %

^a^2003

^b^2008

**Fig. 4:**
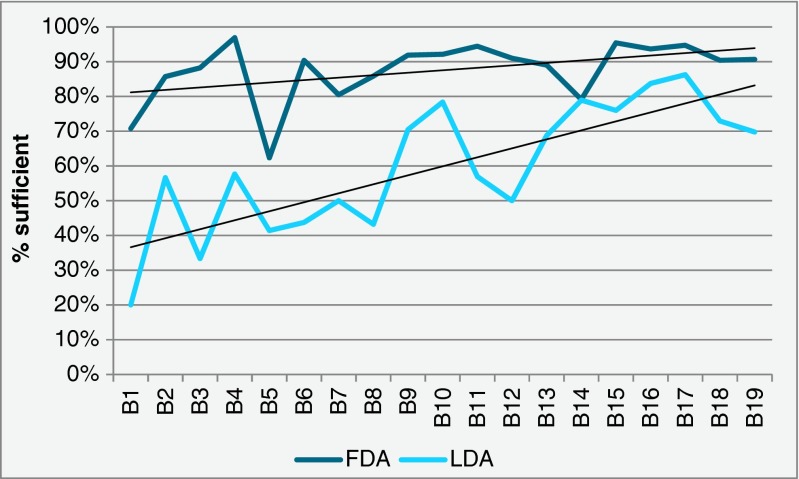
Proportion of sufficient HER2 IHC test results in 19 runs for FDA approved and laboratory-developed assays (LDA). See text for details

**Fig. 5:**
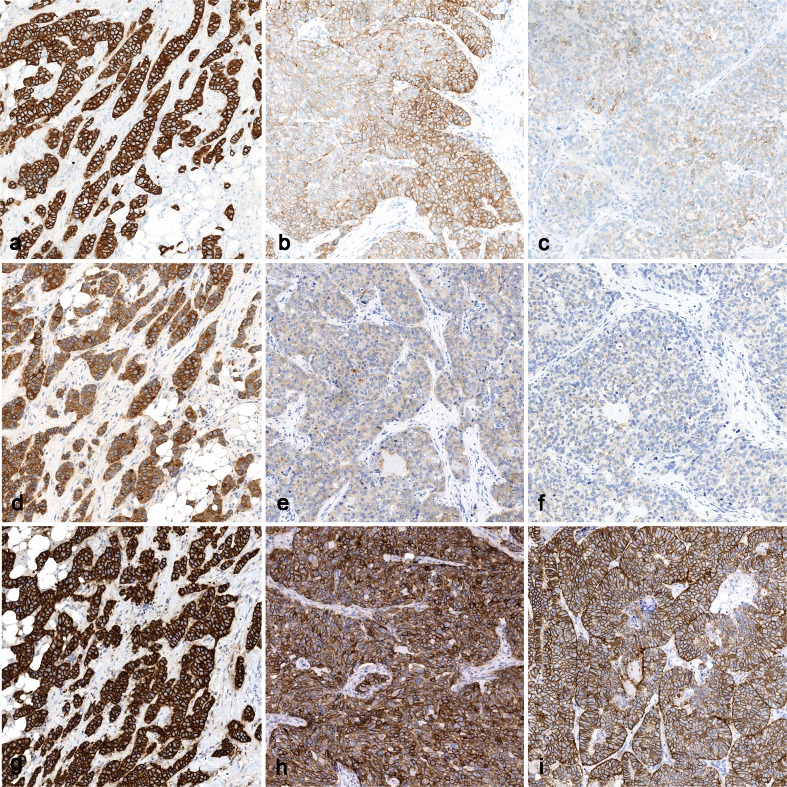
Staining for HER2 protein. **a**–**c** Optimal staining of 3+ staining of HER2 in gene-amplified ductal breast carcinoma (**a**), 2+ staining of HER2 in gene-amplified ductal breast carcinoma (**b**), and 1+ staining of HER2 in gene-unamplified ductal breast carcinoma (**c**). **d**–**f** Insufficient (too weak staining) of the same tumors as in **a**–**c**, using a laboratory-developed protocol: still a 3+ staining of the carcinoma in (**d**), but a 1+ staining of the carcinoma in (**e**) and 0 staining of the carcinoma in (**f**). **g**–**i** Insufficient (too strong staining) of the same tumors as in **a**–**c**, using a laboratory-developed protocol: strong 3+ staining in (**g**) and (**h**), false positive reaction in (**i**) (×200)

In tests for HER2 IHC, included in 19 runs during 2005–15, the US Food and Drug Administration (FDA) approved kits gave sufficient results in a high proportion throughout the period, close to 90 % in the latest runs. In contrast, laboratory-developed assays most often gave poor results in the first runs and slowly improved but is still below the level of the FDA and Conformité Européene (CE) in vitro diagnostics (IVD) approved (Fig. 4). However, also variation of the pass-rates between different FDA-approved kits has been demonstrated. Examples of optimal and insufficient HER2 stains are illustrated in Fig. 5.

**Fig. 6:**
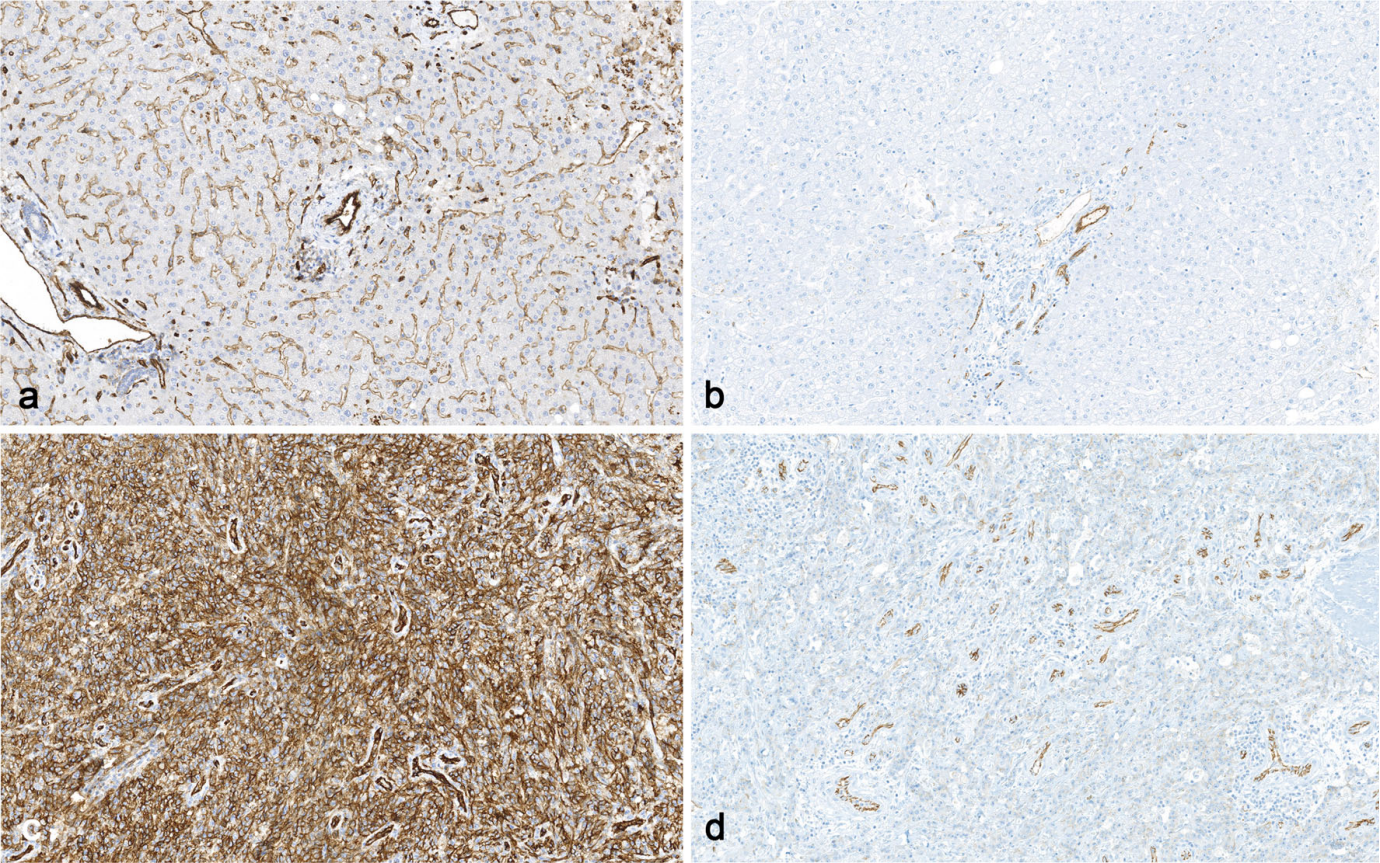
Staining for CD31. **a** Optimal staining of normal liver, using clone JC70A. Strong staining of the arterial endothelial cells and moderate staining of sinusoidal endothelial cells is seen. **b** Insufficient staining of the same liver as in (**a**) using clone 1A10. The sinusoidal endothelial cells are false negative, while the arterial endothelial cells are still stained. In three runs, 496 CD31 stained slides were assessed, of which 37 were based on clone 1A10, all of which were insufficient. **c** Optimal staining of angiosarcoma using clone JC70. **d** Insufficient staining of the same tumor as in (**c**) using clone 1A10. The tumor is false negative. Only normal endothelial cells with a high level of CD31 expression are demonstrated (×200)

In the general module, PT for almost 90 IHC markers shows a more complex pattern. Improvements were often seen for tests where the laboratories adjusted their protocols according to tailored NordiQC recommendations. Thus, during 2003–2006, challenges for six epitopes (Chromogranin A, Calretinin, CD5, CD15, CD23, and Cytokeratin low molecular weight) were performed three times. A total of 352 laboratories that obtained an insufficient result for one of these tests received specific guidelines on how to improve the performance and participated in a subsequent run. Of the 352 laboratories, 227 (64 %) modified their protocols for the following test for the same epitope, of which a sufficient result was obtained by 167 laboratories (74 %). The remaining 125 laboratories (36 %) did not modify their protocol. Of these, only 22 laboratories (18 %) obtained a sufficient score in a new test. Improvements have also been seen where the use of poorest antibodies/clones have been reduced, typically because the participants have changed to new and better clones and/or the companies have pruned them. This applies for, e.g., Synaptophysin (clone SY38), BSAP (Pax5) (clone 24), and CD31 (clone 1A10) (Fig. 6).

**Fig. 7:**
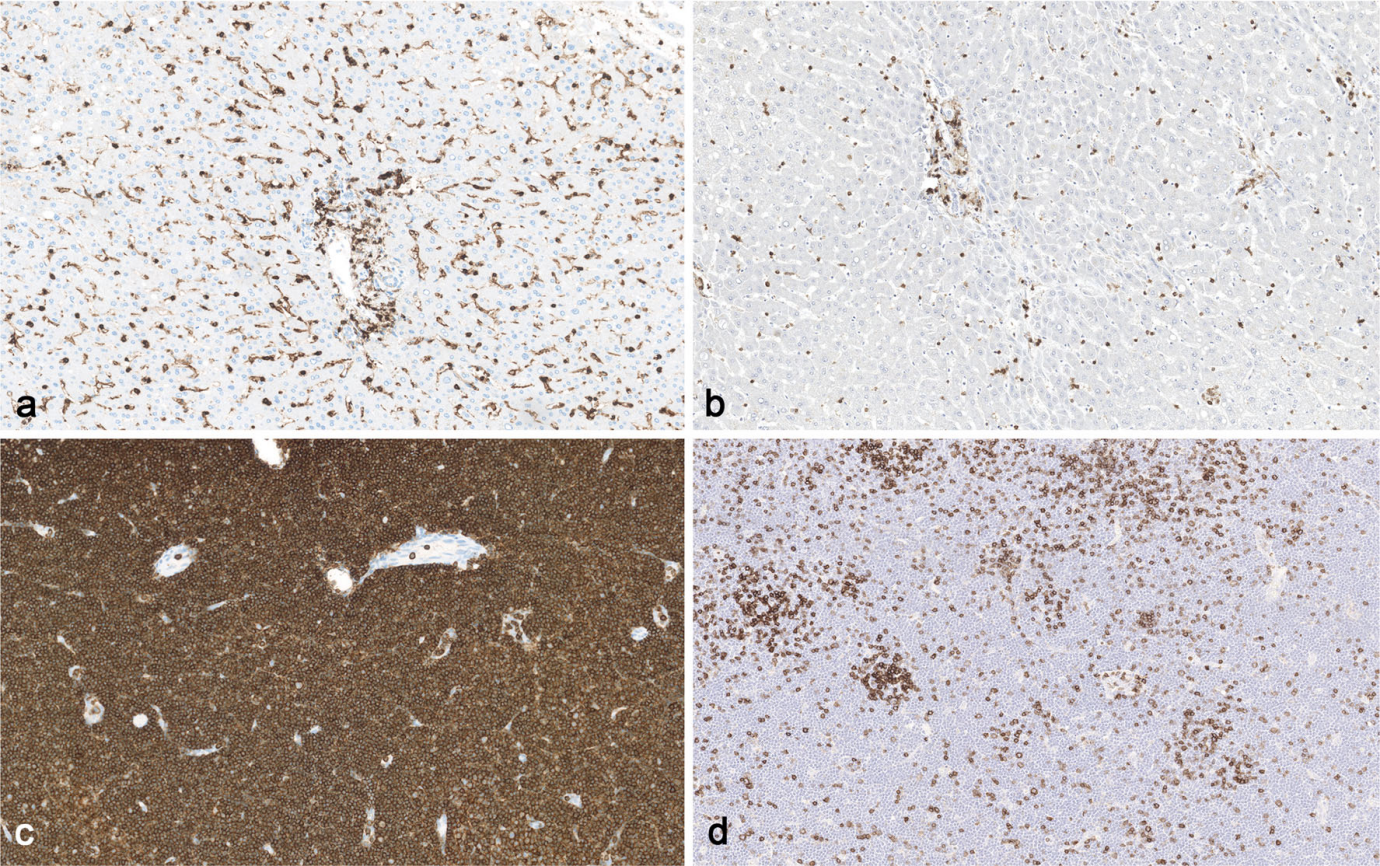
Staining for CD45. **a** Normal liver showing optimal staining, the Kupffer cells and vascular lymphocytes are strongly stained. **b** Same tissue as in (**a**) giving false negative reaction in Kupffer cells with a low level CD45 expression, due to omission of HIER (as recommended by the vendor). **c** B cell chronic lymphatic leukemia optimally stained for CD45 with the same antibody as in (**a**). **d** Same tissue as in (**c**) giving false negative reaction in the neoplastic cells, same protocol as in (**b**) (×200)

**Fig. 8:**
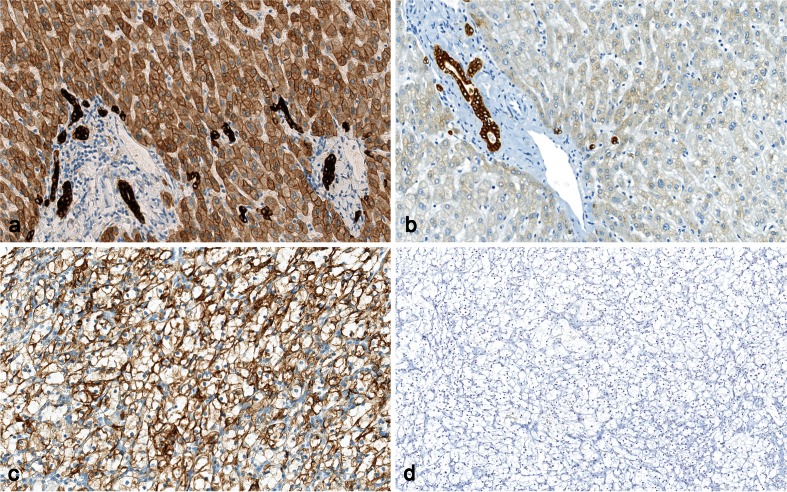
Staining for Pan-cytokeratin (PCK), using clone cocktail AE1/AE3. **a** Normal liver showing optimal staining: strong reaction in bile ducts, moderate reaction in liver cells. **b** Insufficient staining of liver (same tissue as in (**a**)): The liver cells are false negative, due to proteolytic pretreatment (as recommended by the vendor of the primary antibody) instead of HIER (as recommended by NordiQC). **c** Clear cell renal cell carcinoma stained like in (**a**) showing strong membrane-related staining. **d** The same tumor and approximately the same field as in (**c**), same protocol as in (**d**) giving false negative reaction in the tumor (×200)

In contrast little improvement has been realized for suboptimal RTU systems. For example, in the NordiQC assessment for CD45 (run 37, 2013), all protocols based on the RTU system for the clone RP2/18 using the vendor recommended protocol (which specifies omission of epitope retrieval) provided false negative results. In contrast, laboratories who modified the vendor protocol by using HIER significantly improved their results (Fig. 7). Also for tests where vendor recommended protocols giving insufficient results conflict with the NordiQC recommendations, the improvement has often been disappointing. An example is the challenges for Pan-Cytokeratin (Fig. 8).

## Discussion

For many laboratories, calibration and validation of IHC assays for optimal performance remains a difficult task. The implementation of IHC assays is complex, the test process gives an increased workload in the laboratory, and it requires a high level of both technical and diagnostic expertise to interpret the tests performed. This may be difficult to comply with due to limited specialized IHC education and experience in the laboratories. Efforts made to standardize and optimize IHC have typically addressed general principles but not provided the specific data required to help laboratories to evaluate the technical quality of the IHC assays used in routine diagnostics [6, 7]. Owing to the extensive requirements for laboratories to establish technical optimization and validation, the use of RTU antibodies/systems have for many markers gained popularity, but unfortunately some RTU systems will not provide a sufficient result, as shown by NordiQC. Participation in PT programmes provides the laboratories with a tool to overcome some of these difficulties. In addition to PT programmes, various time-limited ring trials have been performed, e.g., a German nationwide PT of breast cancer hormone receptors and HER2 assessment [8]. In this trial, a significant improvement of performance was shown for laboratories participating in more than one trial, which is in line with the NordiQC experience. By publishing important results of the PT programmes and ring trials, all pathology laboratories are offered guidance to optimize their IHC protocols irrespective of participation in a specific programme [6].

Progress in performance for laboratories participating in PT can be hard to evaluate properly. This is in part due to the continuous increase in number of laboratories. Furthermore, the assessment criteria may be adjusted (usually tightened) according to new knowledge, more useful tissues included, or rise of better antibodies and visualization systems, which changes the conception of what is optimal.

Regardless of the cause(s) of insufficient staining results, attention must be focused on the choice of tissue controls used by the laboratory, the EQA programme, and the commercial companies for calibration and validation of the IHC assays. Identification of appropriate tissue controls is an ongoing process. The international ad hoc committee [9, 10] has made considerable contributions to determine the standards for tissue controls to be used by pathology laboratories as well as diagnostic companies developing IHC reagents and equipment. The fact that about 90 % of the insufficient results in the NordiQC PT assessments are characterized by too weak or completely false negative staining reaction clearly indicates that the main challenge to both laboratory-developed and RTU assays is to perform a precise calibration, which can be established only by selecting proper controls with known low amounts of the target epitope [9, 10]. Only by identification and accurate characterization of expected staining patterns in well-defined tissue controls is it possible to evaluate reliably the technical quality and to monitor the impact of changes of analytical variables. For typical qualitative IHC assays, such as CD31 (Fig. 6), CD45 (Fig. 7), Chromogranin A, CDX2, and Cytokeratins (pan-, low, and high molecular weight), it has been shown in the NordiQC programme that precise information regarding the level of technical sensitivity can be made only by interpretation of the staining reaction in tissues with weak expression of the target epitope, whereas misleading conclusions can be made if only tissues with high expression levels are used. This is in line with the cIQc experience [11, 12]. For CDX2, the NordiQC results have been confirmed by comparing different CDX2 antibodies in a large cohort of normal and neoplastic tissues [13]. Only by using pancreas as a CDX2 tissue control and focusing on the ability to demonstrate the small amount of CDX2 in cells of the ductal epithelium can a reliable demonstration of CDX2 be made in neoplasias with low expression levels.

**Fig. 9:**
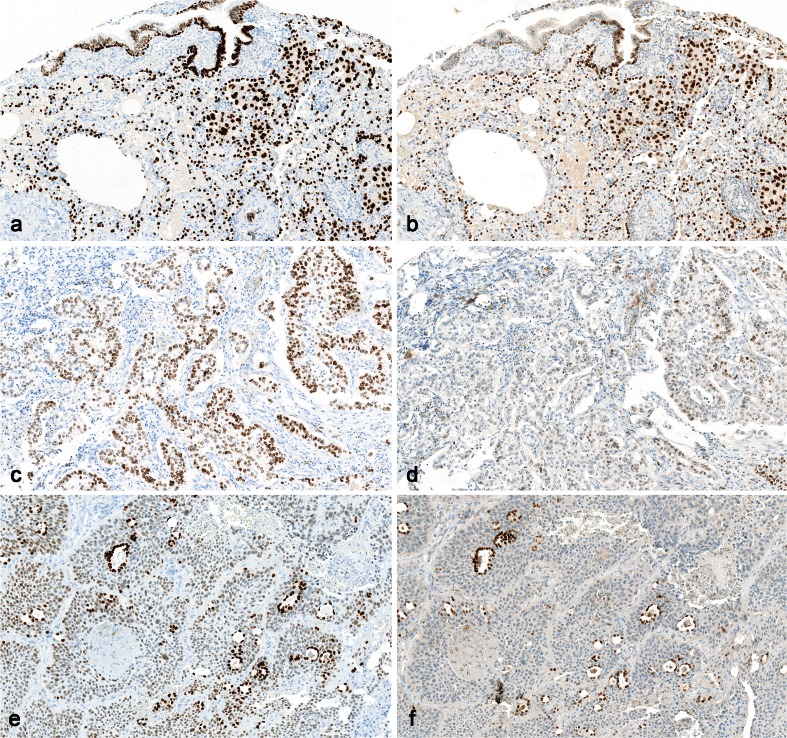
Staining for TTF-1. **a** Poorly differentiated adenocarcinoma (*right*) showing strong staining. A normal bronchus (*top*) showing strong staining of basal cells and moderate staining of luminal cells. The staining based on clone SPT24. Clone SP141 gives the same reaction. **b** Same field as in (**a**) showing moderate staining of the tumor, weak staining of the basal cells and negative reaction of the luminal cells. The staining is based on clone 8G7G3/1 and a carefully calibrated protocol to provide the best possible technical signal-to-noise ratio. Clone MX011 gives the same staining reaction. **c** Lung adenocarcinoma stained with the same antibody and protocol as in (**a**). Moderate staining of all tumor cells. **d** Same field as in (**c**), same antibody and protocol as in (**b**). The tumor is false negative. Normal pneumocytes are stained. **e** Lung squamous cell carcinoma (which was strongly positive for p40) stained with the same antibody and protocol as in (**a**). Moderate staining of tumor cells. Note the strongly stained pnenumocytes. **f** Same field as in (**e**) stained with the same antibody and protocol as in (**a**). The tumor is negative for TTF-1 (few nuclei equivocally positive). Note the pneumocytes stained only slightly weaker than in (**e**) (×200)

Selection of sensitive antibodies and optimization of protocols to the best signal-to-noise ratio may in some cases hamper the “diagnostic specificity.” E.g., in lung cancer, reports have found that thyroid transcription factor-1 (TTF-1) clone 8G7G3/1 was positive in 1 % of squamous cell carcinomas, and 65–77 % of lung adenocarcinomas, whereas TTF-1 clone SPT24 was positive in 17 % of squamous cell carcinomas and 72–84 % of adenocarcinomas [14]. In order to avoid the TTF-1 staining of squamous cell carcinomas, WHO recommends usage of clone 8G7G3/1 [15]. However, this recommendation may conflict with the principle of optimizing staining reactions to the best signal-to-noise ratio which include selection of the most sensitive and specific clones and carefully calibrating the dilution on low expressing cells in normal tissues (Fig. 9). Setting up appropriate antibody panels may be a better alternative to the use of less sensitive antibodies or suboptimal protocols which will impede standardization of IHC.

Recently and looking toward the future, IHC will be providing a window onto the molecular alterations which underlie cancers. This has been designated as “Next generation IHC” [16]. Due to genetic tumor changes, proteins may be lost (e.g., mismatch repair proteins, E-cadherin, INI1), over-expressed (e.g., p53, HER-2, bcl-2), or antigenically changed (e.g., ALK, BRAF, IDH1), which may be revealed by IHC. As these tests are “stand-alone” assays, they make high demands on the IHC standardization.

In assays where semiquantitative analyses are to be carried out (e.g., HER2, estrogen receptor protein, Ki67), the problems with inter- and intra-laboratory reproducibility may be overcome by using digital image analysis (DIA) [17]. However, in order to introduce DIA in the diagnostic work, standardization and EQA becomes even more important. DIA can also be implemented in PT assessment to strengthen the manual procedure. Thus, in a NordiQC study, DIA of HER-2 stained slides of breast cancers could be used to define precise levels of membrane connectivity to distinguish between optimal and suboptimal staining reactions, allowing for better calibration of the immunoassays [18].

IHC PT programmes based on expert panel-based qualitative assessment systems, which work internationally and/or publish their results in English, include United Kingdom National External Quality Assessment Scheme for Immunocytochemistry (UK NEQAS ICC), Canadian Immunohistochemical Quality Control (cIQc), The Royal College of Pathologists of Australasia, Quality Assurance Programmes (RCPAQAP), and NordiQC. These programmes use different approaches in evaluating the performance of individual participating laboratories but are all rooted in a comparative method in which a judgment is rendered by a panel of expert assessors, striving to achieve consistency and accuracy in the operation of clinical laboratories with the ultimate goal of improved patient safety. The time has come for these and other PT programmes to joining forces for common standards in IHC. Initiatives have currently been taken to construct an International Quality Network for Pathology (IQN Path, www.iqnpath.org) aimed to delivering improved clinical implementation of tissue-based biomarkers through multi-stakeholder cooperation, exchange expertise between key opinion leaders in the field, pool resources to quickly establish recommendations for new biomarker adoption, establish benchmarks and best practice, coordinate interaction between international experts and different stakeholders involved in quality assessment thereby supporting faster adoption of new biomarkers and technology, and promote EQA/PT by creating compelling evidence to inform and lead policy development, identifying trends and emerging needs in the field creating a stronger voice for EQA providers (www.iqnpath.org).
